# Beta-hydroxybutyrate, an endogenous NLRP3 inflammasome inhibitor, attenuates anxiety-related behavior in a rodent post-traumatic stress disorder model

**DOI:** 10.1038/s41598-020-78410-2

**Published:** 2020-12-10

**Authors:** Takehiko Yamanashi, Masaaki Iwata, Midori Shibushita, Kyohei Tsunetomi, Mayu Nagata, Naofumi Kajitani, Akihiko Miura, Ryoichi Matsuo, Tsuyoshi Nishiguchi, Takahiro A. Kato, Daiki Setoyama, Yukihiko Shirayama, Ken Watanabe, Gen Shinozaki, Koichi Kaneko

**Affiliations:** 1 Department of Neuropsychiatry, Faculty of Medicine, Tottori University, 86 Nishi-cho, Yonago, Tottori 683-8503 Japan; 2 Department of Psychiatry, University of Iowa Carver College of Medicine, Iowa City, IA USA; 3 Department of Neuropsychiatry, Graduate School of Medical Sciences, Kyushu University, Fukuoka, Japan; 4 Clinical Laboratories, Kyushu University Hospital, Fukuoka, Japan; 5 Department of Psychiatry, Teikyo University Chiba Medical Center, Ichihara, Japan; 6 Watanabe Hospital, Tottori, Japan

**Keywords:** Post-traumatic stress disorder, Neuroimmunology

## Abstract

Accumulating evidence suggests that elevated inflammation contributes to the pathophysiology of post-traumatic stress disorder (PTSD) and that anti-inflammatory drugs might be a new treatment strategy for PTSD. It has been reported that beta-hydroxybutyrate (BHB), one of the main ketone bodies produced, can have an anti-inflammatory and antidepressant effect. Here, we investigated the potential anti-anxiety and anti-inflammatory effects of BHB using a rodent PTSD model, induced by single prolonged stress (SPS). Male, Sprague–Dawley rats were employed in this study. Repeated administration of BHB attenuated SPS-induced anxiety-related behaviors evaluated by the elevated plus maze test. SPS increased the serum levels of TNF-α and IL-1β. In contrast, BHB administration partially attenuated the increase of serum TNF-α. These findings demonstrate that BHB exerts its anxiolytic effects, possibly by inhibiting systemic TNF-α. Hence, BHB may be a novel therapeutic candidate for the treatment of PTSD.

## Introduction

Post-traumatic stress disorder (PTSD) is a mental disorder that may occur after experiencing traumatic events such as war, disaster or violence^1,2^. Symptoms of PTSD include the avoidance of trauma-related stimuli, intrusion symptoms, negative alterations in cognitions and mood, and hyperarousal. In addition, PTSD is associated with anxiety disorders, mood disorders, and with substance abuse^1^. Although patients with PTSD are treated with serotonin reuptake inhibitors (SSRIs), approximately 20–50% of them exhibit little or no improvement^3^. Thus, the investigation for novel pharmacological therapies is a high priority.

Accumulating evidence suggests that elevated inflammation contributes to the pathophysiology of PTSD^4–6^. Recent reports showed that hippocampal mRNA of interleukin-1beta (IL-1β) and tumor necrosis factor-alpha (TNF-α) were increased in a rat model of PTSD, and that substances which ameliorated PTSD-related behavior attenuated elevation of hippocampal mRNA of inflammatory cytokines^7,8^. Furthermore, a recent meta-analysis showed that some inflammation-related biomarkers, including TNF-α and IL-1β, were higher in subjects with PTSD than in healthy subjects^4,6^. Hence, anti-inflammatory drugs or substances have been proposed as a new treatment strategy for PTSD^6^.

Beta-hydroxybutyrate (BHB) is a ketone body that supports mammalian survival during energy-deficit states^9,10^. It has been shown that BHB reduces inflammatory cytokine release, mediated by the leucine-rich repeat, pyrin-domain-containing 3 (NLRP3) inflammasome^11^. We have previously demonstrated that BHB exerts an antidepressant effect in rodent models of depression by inhibiting neuroinflammation^12,13^. Furthermore, studies using other rodent models have also demonstrated that BHB attenuated schizophrenia- and depression- like behavior^14,15^. Therefore, we hypothesized that BHB could possess a potentially therapeutic effect on PTSD-related behaviors.

To evaluate the possible beneficial effects of BHB on PTSD, we used a single prolonged stress (SPS) model that, in rodents, promotes hallmark features of PTSD: long-lasting traumatic memories, persistent anxiety, and hormonal abnormalities^16,17^. In the present study, the primary objective was to investigate whether BHB affects SPS-induced anxiety-like behavior, representing PTSD abnormalities, while the secondary objective was to investigate the relationship between the systemic immune mechanism and PTSD.

## Results

### Subcutaneous single administration BHB increased blood BHB levels

**Figure 1:**
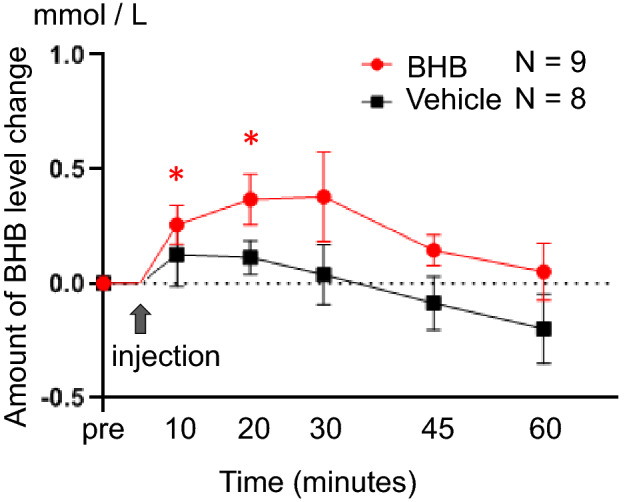
Elevated levels of blood BHB after subcutaneous administration of BHB. Levels of blood BHB were measured after subcutaneous injection of BHB. BHB concentration significantly increased 10–20 min after injection compared to the one before BHB injection (paired t-test; 10 min: t = − 3.00, df = 8, p = 0.017, 20 min: t = − 3.32, df = 8, p = 0.011), and gradually decreased thereafter. BHB concentration did not significantly increase after PBS injection. The degree of change in BHB was calculated as follows: (the level of BHB at each time point)—(the level of pre-injection BHB). Data are shown using mean value and standard error. Paired t-test was performed to compare blood BHB level at pre-injection and at each time point. n = 9/BHB injection group and n = 8/Vehicle injection group. BHB: beta-hydroxybutyrate, * p < 0.05.

We assessed the pre-injection blood concentration of BHB and then again at 10, 20, 30, 45 and 60 min after a single subcutaneous administration of BHB. The concentration of blood BHB increased significantly 10–20 min after BHB injection compared to the one before BHB injection (paired t-test; 10 min: t = − 3.00, df = 8, p = 0.017, 20 min: t = − 3.32, df = 8, p = 0.011, n = 9/Vehicle group, n = 8/BHB group), but not after PBS injection (Fig. 1).

### SPS induced anxiety-related behavior in the EPM

**Figure 2:**
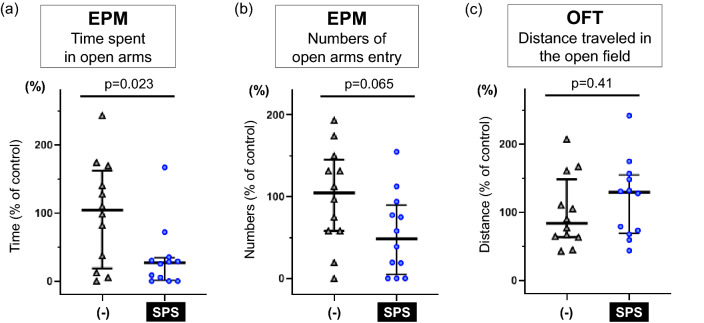
SPS induces anxiety-like behavior in the EPM. (**a**) Time spent in the open arms of the EPM is shown. Exposure to SPS significantly reduced time spent in the open arms (median [IQR]; non SPS: 104.2 [31.1–147.5], SPS: 26.9 [4.0–31.5], Mann–Whitney’s U-test; W = 111, p = 0.023). (**b**) Number of open-arm entries in the EPM are shown. SPS tended to decrease the number of open-arm entries (median [IQR]; non SPS: 104.7 [58.1–136.0], SPS: 48.4 [14.1–81.5], Mann–Whitney’s U-test; W = 104, p = 0.065). (**c**) Distance travelled in the OFT is shown. Exposure to SPS did not change distance travelled (median [IQR]; non SPS: 83.6 [64.1–122.9], SPS: 129.2 [71.8–150.2], Mann–Whitney’s U-test; W = 57, p = 0.41). Data are shown using % of controls. Reference control is the (-) group. Data are presented as scatter plots including median and interquartile range. Mann–Whitney’s U-test was performed to compare two groups. n = 12/group. (-): non-SPS group, EPM: elevated plus maze test, OFT: open field test, SPS: single prolonged stress.

First, we confirmed that the SPS-induced PTSD model in rats, produced anxiety-related behavior, consistent with previous studies^8,18–21^. In the EPM, SPS significantly reduced time spent in the open arms (W = 111, p = 0.023, n = 12/group) (Fig. 2a) and tended to decrease the number of open arms entries (W = 104, p = 0.065, n = 12/group) (Fig. 2b). However, SPS did not change locomotor activity in the OFT (W = 57, p = 0.41, n = 12/group) (Fig. 2c). The results of other behaviors are shown in Supplemental Fig. 1.

### Peripheral BHB treatment attenuated anxiety-related behavior in a rodent PTSD model

**Figure 3:**
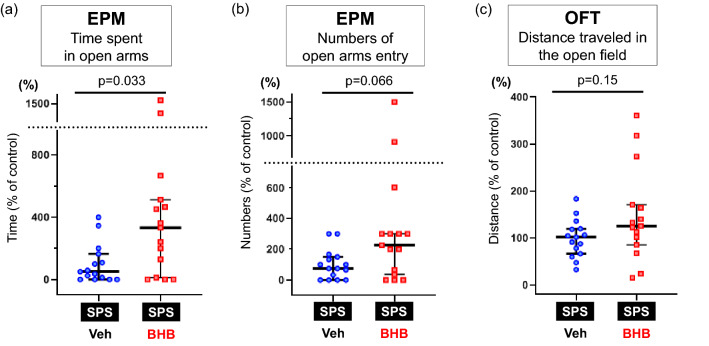
BHB treatment attenuates SPS-induced anxiety-like behavior. (**a**) The time spent in the open arms of the EPM is shown. Rats exposed to BHB had significantly increased time spent in the open arms of the EPM in SPS-induced PTSD rats (median [IQR]; SPS + Veh: 50.0 [6.3–136.5], SPS + BHB: 333.3 [70.5–489.6], Mann–Whitney’s U-test; W = 61.5, p = 0.033). (**b**) Number of open-arm entries in the EPM are shown. BHB tended to increase the number of open-arm entries in SPS-induced PTSD rats (median [IQR]; SPS + Veh: 75.0 [16.7–141.7], SPS + BHB: 225.0 [52.1–300.0], Mann–Whitney’s U-test; W = 68.5, p = 0.066). (**c**) Distance traveled in the OFT is shown and was unchanged after BHB treatment (median [IQR]; SPS + Veh: 101.7 [72.5–119.2], SPS + BHB: 125.9 [93.6–167.6], Mann–Whitney’s U-test; W = 77, p = 0.15). Data are shown using % of controls. Reference control is the SPS + Veh group. The data are presented as scatter plots including median and interquartile range. Mann–Whitney’s U-test was performed to compare two groups. n = 15/group. EPM: elevated plus maze test, OFT: open field test, SPS: single prolonged stress, Veh: Vehicle (PBS), BHB: beta-hydroxybutyrate, n = 15 The data are presented as scatter plots including median and interquartile range.

Next, we evaluated the effect of BHB administration in rats exposed to a SPS. In the EPM, repeated peripheral injection with BHB significantly increased the time spent in the open arms (W = 61.5, p = 0.033, n = 15/group) (Fig. 3a) and tended to increase the number of open arms entries (W = 68.5, p = 0.066, n = 15/group) of rats exposed to the SPS (Fig. 3b). However, locomotor activity in the OFT was not changed by BHB administration (W = 77, p = 0.15, n = 15/group) (Fig. 3c). The results of other behaviors are shown in Supplemental Fig. 2.

### BHB alone did not change anxiety-related behavior

**Figure 4:**
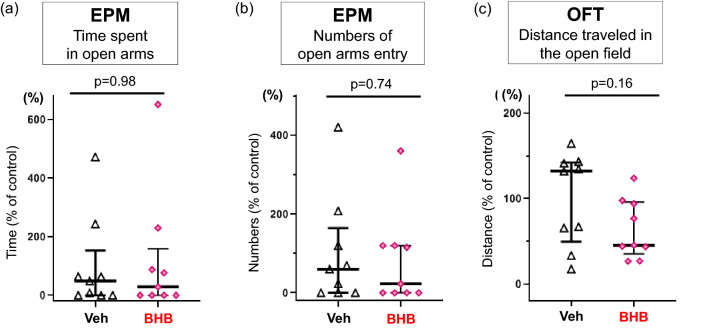
BHB alone does not change anxiety-like behavior or locomotor activity. (**a**) The time spent in the open arms of the EPM is shown. BHB administration did not change the time spent in the open arms (median [IQR]; Veh: 48.7 [0.0–63.8], BHB: 29.2 [0.0–67.7], Mann–Whitney’s U-test; W = 41, p = 0.98). (**b**) Number of open- arm entries in the EPM is shown. BHB administration did not change the number of open-arm entries (median [IQR]; Veh: 60.0 [0.0–120.0], BHB: 23.1 [0.0–120.0], Mann–Whitney’s U-test; W = 44.5, p = 0.74). (**c**) Distance traveled in the OFT is shown. BHB treatment did not change distance traveled (median [IQR]; Veh: 132.1 [65.9–141.5], BHB: 45.5 [43.8–94.1], Mann–Whitney’s U-test; (W = 57, p = 0.16). Data are shown using % of controls. Reference control is the Veh group. The data are presented as scatter plots including median and interquartile range. Mann–Whitney’s U-test was performed to compare two groups. n = 9/group. EPM: elevated plus maze test, OFT: open field test, Veh: Vehicle (PBS), BHB: beta-hydroxybutyrate, n = 9.

To understand how BHB itself affects anxiety-related behavior, we evaluated rats after 2 weeks of BHB administration without being exposed to the SPS paradigm. BHB alone did not change time spent in the open arms (W = 41, p = 0.98, n = 9/group) (Fig. 4a) or the number of open arms entries (W = 44.5, p = 0.74, n = 9/group) (Fig. 4b) in the EPM, nor locomotor activity in the OFT (W = 57, p = 0.16, n = 9/group) (Fig. 4c). The results of other behaviors are shown in Supplemental Fig. 3.

### BHB administration attenuated the SPS-induced elevation in TNF-α

**Figure 5:**
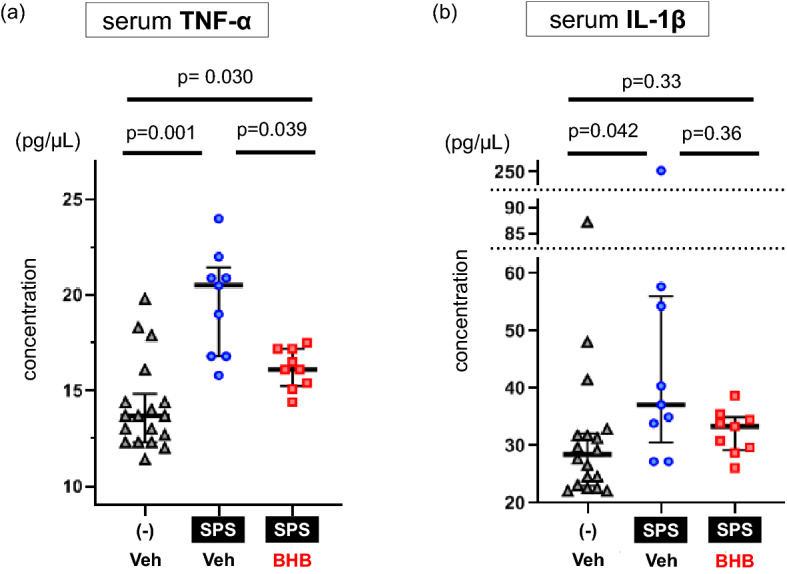
BHB reduces SPS-induced increase in serum inflammatory cytokine levels. (**a**) Serum TNF-α levels are shown (median [IQR]; non-SPS + Veh: 13.7 [12.4–14.4], SPS + Veh: 20.5 [16.8–20.9], SPS + BHB: 16.1 [15.4–17.2], Kruskal–Wallis test; X2 = 17.265, df = 2, p = 0.0002). SPS exposure increased levels of TNF-α in the blood (Mann–Whitney’s U-test; W = 11, p = 0.001). Chronic BHB treatment decreased levels of TNF-α in the blood in SPS-induced PTSD rats (Mann–Whitney’s U-test; W = 69, p = 0.039). (**b**) Serum IL-1β levels are shown (median [IQR]; non-SPS + Veh: 28.5 [23.5–31.8], SPS + Veh: 37.1 [33.9–54.3], SPS + BHB: 33.4 [29.7–34.5], Kruskal–Wallis test; X2 = 7.503, df = 2, p = 0.024). SPS increased levels of blood IL-1β (Mann–Whitney’s U-test; W = 34, p = 0.042). Chronic BHB treatment did not significantly decreased levels of IL-1β in the blood of rats exposed to SPS (Mann–Whitney’s U-test; W = 49.5, p = 0.33). The data are presented as scatter plots with median and interquartile range. Kruskal–Wallis test and Mann–Whitney U tests were performed to compare each group. p-values were corrected by Bonferroni method. TNF-α: Tumor necrosis factor-alpha, IL-1β: Interleukin-1 beta, SPS: single prolonged stress, Veh: Vehicle (PBS), BHB: beta-hydroxybutyrate, n = 18 (non SPS + Veh group) or 9 (SPS + Veh group and SPS + BHB group).

We evaluated changes in pro-inflammatory cytokine levels induced by SPS exposure and after BHB without behavioral testing. Kruskal–Wallis test showed a significant difference of serum TNF-α levels (X2 = 17.265, df = 2, p = 0.0002, n = 18/non-SPS + PBS group, n = 9/SPS + PBS group, n = 9/SPS + BHB group) and IL-1β levels (X2 = 7.503, df = 2, p = 0.024, n = 18/non-SPS + PBS group, n = 9/SPS + PBS group, n = 9/SPS + BHB group) among the three groups. As expected, SPS significantly elevated serum TNF-α levels (W = 11, p = 0.001). However, BHB administration significantly attenuated this elevation (W = 69, p = 0.039) (Fig. 5a). Levels of serum TNF-α in BHB-administrated PTSD rats were higher than in the control group (W = 32, p = 0.030). Serum IL-1β levels were also significantly elevated by SPS exposure (W = 34, p = 0.042). BHB did not significantly attenuate this elevation (W = 58.5, p = 0.36) (Fig. 5b). Further, serum IL-1β level in BHB-administered PTSD rats were not significantly different from those in the control group (W = 49.5, p = 0.33).

### Other information

All rats were drug or test naïve prior to treatment or testing. Mean body weight of rats just before treatment or testing was 265.9 g (standard deviation: 27.1, n = 125). No important adverse event was found throughout the study.

## Discussion

In the present study, we observed that BHB administration produced anxiolytic effects in a rat model of SPS-induced PTSD. We also examined the influence of BHB on elevated serum TNF-α and IL-1β induced by SPS, and found that BHB attenuated SPS-induced increases in TNF-α. This study provides new evidence suggesting that BHB exerts anti-anxiety effects, and that BHB may reduce systemic inflammation in rodents exposed to a SPS-induced PTSD model.

BHB is one of ketone bodies synthesized in the liver of mammals. BHB serves as an energy source for the brain when carbohydrate access is limited, such as in starvation^9^. Recent reports have implicated BHB in various neurodegenerative conditions such as Alzheimer’s disease, Parkinson’s disease, and Huntington’s disease. Specifically, BHB has been shown to prevent striatal histone deacetylation, improve mitochondrial respiration, inhibit amyloid-β aggregation, and attenuate brain atrophy^22–24^. Moreover, rats receiving intravenous administration of BHB showed reduced damage after focal cerebral ischemia, possibly by decreasing radical oxygen production^25^. Additionally, several studies have elucidated the involvement of BHB in stress-related psychiatric disorders. One study reported that BHB is associated with symptom severity in patients with depression^26^. Using a chronic unpredictable stress (CUS)-induced depression model and an acute stress model in rats, we previously found that repeated BHB administration exerted antidepressant-like effects. Specifically, we observed that BHB attenuated the effects of stress exposure and promoted a decrease in immobility time in the forced swim test (FST), increased sucrose preference in the sucrose preference test, increased time spent in the open arms of the EPM, and decreased latency to feed in the novelty suppressed feeding test. Additionally, we showed that BHB exerts its effects, at least in part, through the reduction of pro-inflammatory cytokines within the hippocampus^12^. Other work has shown that CUS induced-depressive behaviors in mice are ameliorated by repeated BHB administration through increasing histone3-lysine9-β-hydroxybutyration and brain-derived neurotrophic factor (BDNF)^15^. In addition, we recently reported that 3-weeks of BHB administration into the prefrontal cortex (PFC) decreased immobility time in the FST and attenuated the elevation of TNF-α within the PFC^13^. These findings suggest that BHB participates in the pathophysiology of stress-related psychiatric disorders, and that BHB might present a novel treatment option for patients. However, until now, no published study has assessed BHB using PTSD animal models or PTSD subjects.

We then evaluated the anxiolytic effects of BHB by using a SPS procedure. The SPS paradigm is an animal model of PTSD with high face, construct, and predictive validity^17^. SPS consists of three different types of stressors—psychological stress (immobilization), physiological stress (forced swimming), and pharmacological stress (diethyl ether)^27^. It has been reported that SPS-induced behavioral changes are observed after seven days, but not one day, of exposure to the procedure, suggesting that behavioral changes promoted by SPS are time-dependent^17,27^. This behavioral change feature parallels PTSD-like symptoms because those who experience multiple traumas, or a trauma early in life, are more susceptible to developing PTSD following a subsequent traumatic event^17^. We confirmed that rats exposed to SPS exhibited anxiety-related behavioral changes, including decreased time spent in open arms and a trend of decreased numbers of open arm entries in the EPM (Fig. 2a,b). These results are consistent with past studies using SPS procedures^8,18–20^. Moreover, our results indicate that chronic BHB administration prevented the development of these SPS-induced anxiety-related behaviors (Fig. 3a,b), even though BHB itself did not change the behavioral tendencies in the EPM (Fig. 4a,b). Behavioral alterations induced by SPS or BHB were observed without changes in locomotor activity in the OFT (Figs. 2c, 3c, 4c), thus these behavioral changes are considered changes in the anxiety state of rodents and not as ambulatory side effects^28–30^. Our results suggest that BHB itself does not exert anti-anxiety effects but, consistent with our previous report, BHB can attenuate SPS-induced anxiety^12^.

We also found that serum levels of TNF-α and IL-1β were elevated two weeks after SPS (Fig. 5a,b). Several human studies have shown elevated plasma/serum levels of TNF-α in PTSD subjects^5,6^ and a recent meta-analysis showed a significant increase in serum TNF-α level in PTSD subjects compared with healthy controls^4^. In a cohort of trauma-exposed Vietnam War veterans, it was found that there was a correlation between PTSD severity and elevated serum TNF-α levels^31^. IL-1β has also been investigated in PTSD subjects or PTSD animal models^32^ and a meta-analysis revealed elevated levels of serum IL-1β in subjects with PTSD^4,33^. Our present results, which show elevated levels of serum TNF-α and IL-1β after SPS exposure, are consistent with previous studies^7^. In addition, we demonstrated that repeated BHB administration attenuated serum TNF-α elevation in rats exposed to SPS partially, but not completely (Fig. 5a). One previous study using a SPS-induced PTSD model, showed that administration of oleuropein, a compound in olive leaves, attenuated SPS-induced behavior and prevented elevations of serum TNF-α and IL-1β^7^. This study also demonstrated that oleuropein inhibited SPS-induced increases of TNF-α and IL-1β mRNA within the hippocampus^7^. Another study showed similar results for ibuprofen when used as a therapeutic in PTSD rodent models^8^. Unfortunately, these studies showing cytokine changes cannot clarify how cytokines contribute to the pathophysiology of PTSD. Furthermore, there is insufficient clinical evidence highlighting the effect of anti-inflammatory drugs for PTSD^6^. However, it is possible that drugs or substances which have anti-inflammatory effect, may present a novel treatment option for patients with PTSD. These findings suggest that BHB exerts anti-anxiety effects in PTSD rodent models through systemic anti-inflammatory effects.

Evidence has shown that BHB exerts anti-inflammatory effects via suppressing the activation of the NLRP3 inflammasome^34–36^. The NLRP3 inflammasome is composed of NLRP3, adapter protein ASC, and pro-caspase-1. NLRP3 is a cytosolic pattern-recognition receptor. In the central nervous system, NLRP3 is expressed in microglia and is activated to form the inflammasome, which in turn promotes maturation of inflammatory cytokines^37,38^. Thus, it has been suggested that NLRP3 plays a key role in mood disorders associated with neuroinflammation^9–43^. Although there is no evidence showing that NLRP3 plays a role in the pathology of PTSD, BHB might exert its anti-inflammatory effects in a rodent PTSD model via NLRP3 inflammasome modulation.

There are several limitations in the present study. First, our data is based on male rats only. The prevalence, severity, and burden of PTSD is higher in women than in men^44^. Thus, a future study, using female rats is warranted. Second, although we demonstrate anxiolytic effects of BHB administration, the treatment paradigm used shows a prevention of, rather than a reversal of, SPS-induced maladaptation. Further studies are needed to assess the therapeutic efficacy of BHB in animal models of PTSD. Third, we did not show how BHB affects the central nervous system with regard to its anxiolytic effects nor detail its side effects outside the inflammatory system in a PTSD rat model. Fourth, alternative pathways, such as suppressing oxidative stress via histone deacetylase (HDAC) inhibition, increasing BDNF, and activating hydroxycarboxylic acid receptor 2 (HCAR2), are reportedly involved in the mechanisms underlying BHB activity^15,45,46^ and were not evaluated here. As mentioned above, the attenuation of SPS-induced TNF-α elevation by BHB was partial. Therefore, other mechanisms probably also contribute to BHB anti-anxiety effect. Fifth, we failed to demonstrate that BHB attenuates IL-1β levels after the SPS exposure, with statistical significance (Fig. 5b). Our previous study showed that IL-1β increases only immediately after exposure to a stressor, and then returns to basal levels^41^. It might be difficult to detect changes in IL-1β levels in the absence of an acute stress. In order to better elucidate the mechanisms of BHB through alternate signaling pathways, further studies will be necessary. Sixth, currently BHB injection cannot be applied to clinical settings since there is no human study investigating the effects of BHB injection. Further animal and Phase I studies are needed to assess the safety of BHB direct injection. However, there are several human studies that investigated BHB effects using oral administration of ketone ester^47,48^ which also increase serum BHB levels, and these ketone-related approaches can be possibly applied to a clinical setting.

## Conclusion

BHB treatment exerted anxiolytic effects in a SPS-induced PTSD rat model and attenuated SPS-induced increases in serum levels of TNF-α. These findings suggest that BHB administration may be a useful approach to target the inflammatory mechanisms associated with PTSD.

## Methods

### Animals and housing

Male, wild type, Sprague–Dawley (SD) rats weighing 200–220 g (6 weeks old) which were drug or test naïve were obtained from Charles River Laboratories (Yokohama, Japan). Three rats were housed per cage under a 12-h light/dark cycle (lights on at 7:30 AM and off at 7:30 PM) at a constant temperature (25 °C), with free access to food (Rodent Diet CE-2, CLEA, Tokyo, Japan) and water (tap water). They were allowed to acclimate to the housing environment for 5 days. After the experiment, rats were euthanized by decapitation without drug administration. The experiments were carried out in accordance with the Tottori University Animal Care and Use Committee (IRB approval number h29-Y040) and were carried out in accordance with the institutional guidelines. All efforts were made to minimize the number of animal use and pain suffering or distress to rats.

### Drugs and reagents

We obtained DL-BHB (Tokyo Chemical Industry, Tokyo, Japan) and dissolved it in phosphate-buffered saline (PBS) (NaCl 137 mM, KCl 2.7 mM, Na_2_HPO_4_·12H_2_O 8.1 mM, KH_2_PO_4_ 1.47 mM, pH 7.5) to a concentration of 80 mg/mL (pH of 7.5).

### Experimental design

Experiments were conducted in the following order; 1. (a) Measurement of blood BHB concentration, 2. (b) Behavioral test, 3. (c) Serum cytokine measurement. Totally, 125 rats were used in this study (Supplemental Fig. 4). Rats were randomly divided into each experimental group without any consideration of baseline information.

- Measurement of blood BHB concentration

**Figure 6:**
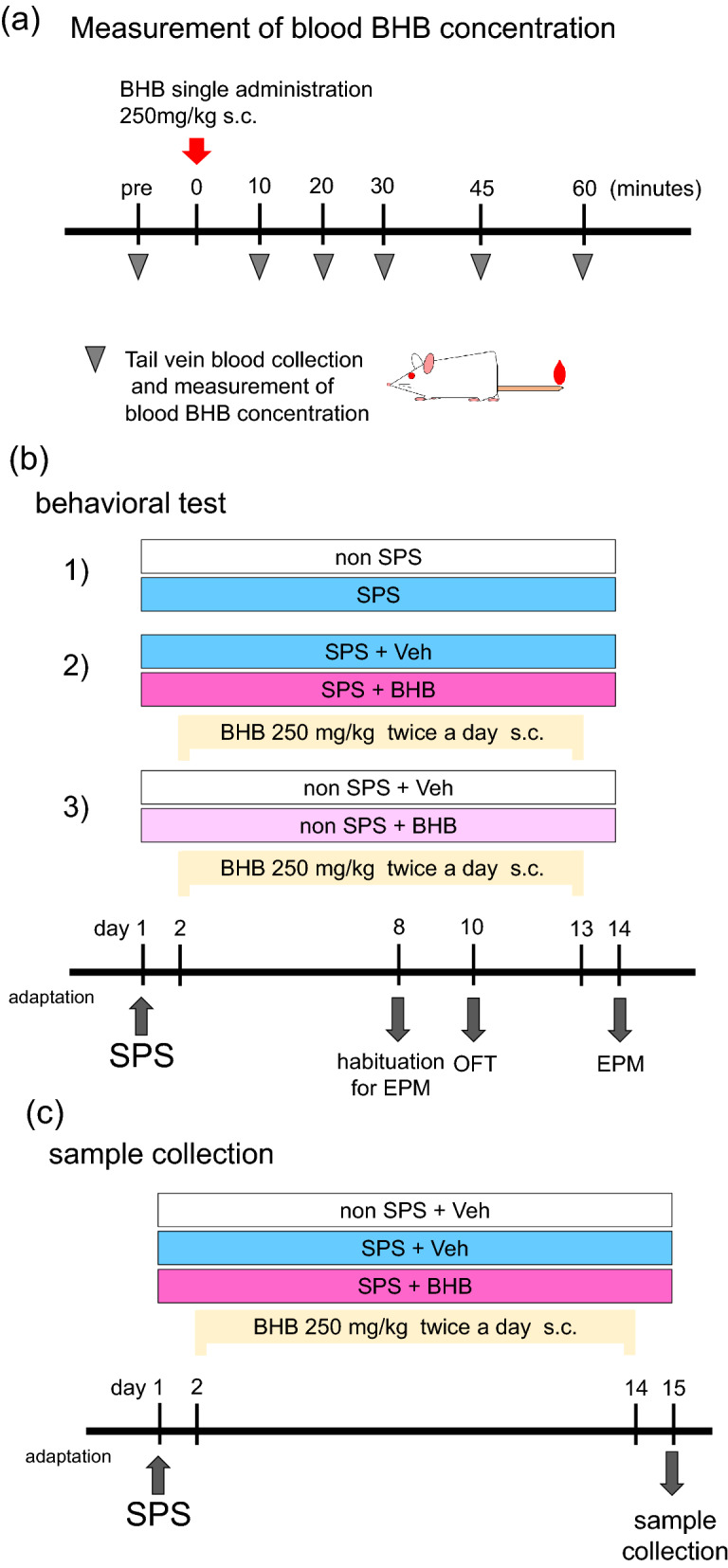
Experimental procedures. (**a**) The procedure for measuring blood BHB concentration is shown. Rats were injected with equal volumes of BHB (250 mg/kg) or PBS. Tail vein blood was collected from the same rats, at the following time points: pre-injection, 10, 20, 30, 45, and 60 min after injection with BHB. (**b**) Schematic representation of the SPS procedure and behavioral tests conducted. The three behavioral experiments performed were as follows: (1) non-SPS (control) vs SPS as a PTSD model (n = 12/group), (2) SPS-induced PTSD without BHB treatment vs SPS-induced PTSD with BHB treatment (n = 15/group), (3) controls without BHB vs controls with BHB (n = 9/group). On day 1, rats were exposed to SPS. From days 2–13, rats were injected with BHB, subcutaneously, twice per day (except for the experiment (1): non-SPS vs SPS). Habituation to the EPM was done on day 8 and the OFT was conducted on day 10, and the EPM on day 14. On behavioral test days, BHB or PBS, was injected following testing. (**c**) Schematic representation of the SPS procedure and blood collection. Three groups were allocated as follows: A non-SPS without BHB administration group (n = 18), a SPS without BHB administration group (n = 9), and a SPS with BHB administration group (n = 9). On day 1, rats were exposed to SPS. From days 2–14, rats were injected with BHB or PBS, twice per day. On day 15, the rats were euthanized, and blood was collected. BHB: beta-hydroxybutyrate, SPS: single prolonged stress, Veh: Vehicle (PBS), EPM: elevated plus maze test. OFT: open field test.

The dose of BHB (250 mg/kg/time) and route of administration (subcutaneous injection) were selected according to our previous study^12^. We measured blood BHB concentration over time to confirm the change of blood BHB concentration after administration of this dose of BHB. Specifically, approximately 10 µL of blood was collected from the same rats, by tail vein draw pre- BHB injection and at various time points (10, 20, 30, 45, and 60 min) after BHB injection (n = 8/PBS injection group, n = 9/BHB injection group) (Fig. 6a). The levels of blood BHB were assayed using a blood glucose/BHB measurement instrument (FreeStyle Precision Neo: Abbott, Japan). The procedure was conducted during the daytime (light cycle). The degree of BHB change was calculated as follows: (the level of BHB at each time point)—(the level of pre-injection BHB).

(b) Behavioral testing timeline

Our study involved three main experiments. The first behavioral experiment was to establish SPS as a PTSD model (non-SPS control vs SPS as a PTSD model, n = 12/group), the second one was to assess the efficacy of a BHB treatment in a PTSD model (SPS as a PTSD model without BHB treatment vs SPS as a PTSD model with BHB treatment, n = 15/group), and the third one was to determine the effects of BHB in stress naïve rats (non-SPS without BHB vs non-SPS with BHB, n = 9/group) (Fig. 6b). The SPS procedure was performed on day 1. From days 2–13, rats were subcutaneously injected with BHB twice a day (around 9:00 am and 5:00 pm) (except for the first experiments with no stress exposure). Rats were habituated to the EPM on day 8. The open field test (OFT) was conducted on day 10, and the EPM on day 14. On behavioral testing days, BHB or PBS were injected following the behavioral tests. BHB or PBS injection was performed in the home cage. Behavioral tests were conducted during daytime in the laboratory. Each experiment was replicated 2–3 times.

(c) Sample collection

Blood was collected from an additional cohort of rats not exposed to behavioral testing: non-SPS without BHB administration group as a control (n = 18), SPS without BHB administration group as a PTSD model (n = 9), and SPS with BHB administration group as a PTSD model with BHB treatment (n = 9). On day 1, rats were given SPS. From days 2–14, rats were injected with BHB or PBS, twice per day in the home cage. On day 15, rats were euthanized and blood was collected. Serum was separated from the blood, and levels of pro-inflammatory cytokines were later measured (Fig. 6c).

### Single prolonged stress (SPS)

SPS was performed as a model of PTSD. On a single day, rats were subjected to a 2-h immobilization, immediately followed by 20 min of forced swimming using a plastic bucket (32 × 47 cm) filled with water (34 cm depth). Rats were given 15 min for recuperation and then subjected to diethyl ether until they were appropriately anesthetized^17,49^. SPS were conducted in the laboratory. Following SPS stressors, the rats were housed three per cage and left undisturbed except during drug administration or behavioral tests.

### Behavioral testing

1. Elevated plus maze test

The EPM apparatus consisted of two open arms (50 × 10 cm) and two closed arms (50 × 10 cm; 30-cm high walls) and an elevated (height of 50 cm) platform. Rats were habituated to the EPM apparatus for 5 min on day 8. This habituation period was needed because some rats fell from the EPM during the initial trial. On day 14, the EPM task was conducted. Rats were placed into the closed arm and allowed to explore the maze for 5 min. Tests were video recorded and later scored by an observer who was blinded to the animal groups. The test was conducted under dark conditions^12^. The maze was cleaned after each trial. The total time spent in open arms and the number of entries into open arms were measured to assess anxiety-related behavior as primary experiment outcomes. The total time spent in closed arms, the number of entries into closed arms, the time spent in the center, the number of staying in the center, the number of protected head dipping, the number of non-protected head dipping, the number of stretched-attend posture, the number of grooming instances, and the number of rearing occurrences were also measured.

(2) Open field test

In the OFT, animals were placed in the open field chamber (90 × 90 × 45 cm) and allowed to explore freely for 10 min. The open field was cleaned after each trial, and all tests were conducted under normal light conditions^13,41^. Tests were recorded, and total moving distance was obtained using a video tracking software system (SMART 3.0: Panlab, U.S.A.) to assess locomotor activity. Time spend in the periphery area, latency to enter the center area (45 cm × 45 cm), the number of center area entries, time spent in the center area, the number of rearing occurrences, and the number of grooming instances were also measured.

### Serum cytokine measurement

Blood was collected and stored at 4 °C overnight. The supernatant was collected and stored at -80 °C until use. Serum levels of TNF-α and IL-1β were measured by using a commercially available ELISA kit (TNF-α; ab236712, IL-1β; ab255730, Abcam, Cambridge, United Kingdom), according to the manufacturer’s protocol. Serum cytokines were evaluated as secondary experimental outcomes.

### Statistical analyses

All statistical analyses were performed with EZR (Saitama Medical Center, Jichi Medical University, Japan), a modified version of R commander designed to add statistical functions frequently used in biostatistics^50^. The sample size of behavioral tests using SPS and BHB (Fig. 3) was calculated using the data from our previous study investigating BHB effects and with a two-tailed significance of α = 0.05 and β = 0.8, while the sample sizes of other experiments were based on previous studies^12,21^. To confirm the blood BHB level elevation, a paired t-test was performed. For comparisons between the two groups (i.e. in behavioral tests), data were analyzed using the Mann–Whitney U test. For comparisons between the three groups, (i.e. in serum cytokine tests), Kruskal–Wallis test and Mann–Whitney U tests with Bonferroni correction were performed to compare each group. The data are presented with scatter plots, median, and interquartile range. P-values < 0.05 were considered significant.

### 3Rs in this study

In this study, it is needed to use rats to investigate the effect of BHB in vivo. We determined the sample size before starting this study to minimize the number of animal use. Although it is possible that several procedures such as SPS, repeated injection, and behavioral tests gave physical or psychological stress to animals, all efforts were made to minimize pain suffering or distress to animals.

## Supplementary information

Supplementary Information 1. **Supplemental Figure 1**: Other behavior comparisons between non-SPS rats and SPS rats. (a) The numbers of closed arms entry of the EPM (median [IQR]; non SPS: 104.1 [87.3–131.7], SPS: 140.5 [110.1–164.7], Mann–Whitney’s U-test; W = 42.5, p = 0.094). (b) The numbers of staying center area of the EPM (median [IQR]; non SPS: 97.5 [45.0–127.5], SPS: 0.0 [0.0–86.3], Mann–Whitney’s U-test; W = 101, p = 0.082). (c) The numbers of open and closed arm entry of the EPM (median [IQR]; non SPS: 109.7 [76.7–130.6], SPS: 105.0 [74.8–132.4], Mann–Whitney’s U-test; W = 70, p = 0.93). (d) Time spent in closed arms of the EPM (median [IQR]; non SPS: 98.5 [82.7–117.9], SPS: 125.6 [118.4–128.9], Mann–Whitney’s U-test; W = 30.5, p = 0.018). (e) Time staying the center area of the EPM (median [IQR]; non SPS: 98.5 [18.75–118.0], SPS: 0.0 [0.0–59.4], Mann–Whitney’s U-test; W = 95.5, p = 0.16). (f) The number of protected head dipping of the EPM (median [IQR]; non SPS: 92.1 [47.7–150.0], SPS: 129.6 [102.3–262.5], Mann–Whitney’s U-test; W = 48.5, p = 0.18). (g) The number of non-protected head dipping of the EPM (median [IQR]; non SPS: 109.6 [17.3–141.4], SPS: 40.4 [0.0–69.2], Mann–Whitney’s U-test; W = 96.5, p = 0.16). (h) The number of stretched-attend posture of the EPM (median [IQR]; non SPS: 50.0 [0.0–200.0], SPS: 175.0 [137.5–525.0], Mann–Whitney’s U-test; W = 35, p = 0.033). (i) The number of grooming of the EPM (median [IQR]; non SPS: 120.0 [0.0–171.4], SPS: 120.0 [64.3–244.3], Mann–Whitney’s U-test; W = 57, p = 0.39). (j) The number of rearing of the EPM (median [IQR]; non SPS: 104.1 [65.5–116.9], SPS: 128.0 [122.2–156.4], Mann–Whitney’s U-test; W = 32, p = 0.022). (k) Time in the periphery area of the OFT (median [IQR]; non SPS: 101.0 [98.8–101.2], SPS: 101.0 [97.6–101.7], Mann–Whitney’s U-test; W = 72, p > 0.99). (l) The number of center area of the OFT (median [IQR]; non SPS: 58.4 [0.0–158.3], SPS: 79.6 [12.5–255.7], Mann–Whitney’s U-test; W = 66, p = 0.75). (m) Time spent in the center area of the OFT (median [IQR]; non SPS: 71.3 [0.0–150.0], SPS: 33.1 [3.2–189.9], Mann–Whitney’s U-test; W = 71, p = 0.98). (n) Latency to the center area of the OFT (median [IQR]; non SPS: 136.1 [40.0–137.1], SPS: 48.9 [30.8–88.6], Mann–Whitney’s U-test; W = 88, p = 0.37). (o) The number of rearing of the OFT (median [IQR]; non SPS: 70.9 [40.8–150.0], SPS: 105.0 [69.2–189.8], Mann–Whitney’s U-test; W = 59.5, p = 0.49). (p) The number of grooming of the OFT (median [IQR]; non SPS: 100.0 [88.9–102.8], SPS: 133.3 [61.1–158.4], Mann–Whitney’s U-test; W = 58.5, p = 0.45). Data are shown using % of controls. Reference control is the (-) group. Data are presented as scatter plots including median and interquartile range. Mann–Whitney’s U-test was performed to compare two groups. n = 12/group. (-): non-SPS group, EPM: elevated plus maze test, OFT: open field test, SPS: single prolonged stress. **Supplemental Figure 2**: Other behavior comparisons between SPS + Veh rats and SPS + BHB rats. (a) The numbers of closed arms entry of the EPM (median [IQR]; SPS + Veh: 103.4 [67.9–134.6], SPS + BHB: 90.6 [67.9–162.5], Mann–Whitney’s U-test; W = 104, p = 0.74). (b) The numbers of staying center area of the EPM (median [IQR]; SPS + Veh: 0.0 [0.0–200.0], SPS + BHB: 0.0 [0.0–500.0], Mann–Whitney’s U-test; W = 93, p = 0.36). (c) The numbers of open and closed arm entry of the EPM (median [IQR]; SPS + Veh: 103.4 [67.7–132.5], SPS + BHB: 126.8 [84.5–175.3], Mann–Whitney’s U-test; W = 86.5, p = 0.29). (d) Time spent in closed arms of the EPM (median [IQR]; SPS + Veh: 100.5 [99.0–102.4], SPS + BHB: 97.2 [80.9–100.6], Mann–Whitney’s U-test; W = 153.5, p = 0.092). (e) Time staying the center area of the EPM (median [IQR]; SPS + Veh: 0.0 [0.0–127.3], SPS + BHB: 0.0 [0.0–393.3], Mann–Whitney’s U-test; W = 99, p = 0.53). (f) The number of protected head dipping of the EPM (median [IQR]; SPS + Veh: 105.9 [62.6–130.6], SPS + BHB: 70.6 [40.2–125.2], Mann–Whitney’s U-test; W = 122, p = 0.71). (g) The number of non-protected head dipping of the EPM (median [IQR]; SPS + Veh: 51.9 [0.0–165.6], SPS + BHB: 207.8 [52.0–366.3], Mann–Whitney’s U-test; W = 71, p = 0.081). (h) The number of stretched-attend posture of the EPM (median [IQR]; SPS + Veh: 100.0 [0.0–128.6], SPS + BHB: 128.6 [66.7–166.7], Mann–Whitney’s U-test; W = 89, p = 0.33). (i) The number of grooming of the EPM (median [IQR]; SPS + Veh: 70.6 [35.3–125.0], SPS + BHB: 100.0 [60.3–141.2], Mann–Whitney’s U-test; W = 84, p = 0.24). (j) The number of rearing of the EPM (median [IQR]; SPS + Veh: 101.3 [88.7–110.8], SPS + BHB: 101.3 [61.6–122.4], Mann–Whitney’s U-test; W = 105, p = 0.77). (k) Time in the periphery area of the OFT (median [IQR]; SPS + Veh: 100.0 [99.8–100.8], SPS + BHB: 100.0 [98.9–100.7], Mann–Whitney’s U-test; W = 124, p = 0.64). (l) The number of center area of the OFT (median [IQR]; SPS + Veh: 35.4 [0.0–219.3], SPS + BHB: 141.5 [0.0–385.6], Mann–Whitney’s U-test; W = 88.5, p = 0.31). (m) Time spent in the center area of the OFT (median [IQR]; SPS + Veh: 18.1 [0.0–173.5], SPS + BHB: 94.5 [0.0–326.7], Mann–Whitney’s U-test; W = 91, p = 0.36). (n) Latency to the center area of the OFT (median [IQR]; SPS + Veh: 100.0 [48.2–100.0], SPS + BHB: 82.9 [17.9–100.0], Mann–Whitney’s U-test; W = 134.5, p = 0.36). (o) The number of rearing of the OFT (median [IQR]; SPS + Veh: 92.3 [69.2–123.4], SPS + BHB: 102.4 [52.4–209.4], Mann–Whitney’s U-test; W = 100, p = 0.62). (p) The number of grooming of the OFT (median [IQR]; SPS + Veh: 94.7 [73.5–135.2], SPS + BHB: 112.5 [72.0–142.1], Mann–Whitney’s U-test; W = 101.5, p = 0.66). Data are shown using % of controls. Reference control is the SPS + Veh group. The data are presented as scatter plots including median and interquartile range. Mann–Whitney’s U-test was performed to compare two groups. n = 15/group. EPM: elevated plus maze test, OFT: open field test, SPS: single prolonged stress, Veh: Vehicle (PBS), BHB: beta-hydroxybutyrate, n = 15 The data are presented as scatter plots including median and interquartile range. **Supplemental Figure 3**: Other behavior comparisons between Veh rats and BHB rats. (a) The numbers of closed arms entry of the EPM (median [IQR]; Veh: 101.9 [95.5–113.2], BHB: 79.2 [40.9–95.5], Mann–Whitney’s U-test; W = 57.5, p = 0.14). (b) The numbers of staying center area of the EPM (median [IQR]; Veh: 0.0 [0.0–0.0], BHB: 0.0 [0.0–0.0], Mann–Whitney’s U-test; W = 40.5, p = 1.00). (c) The numbers of open and closed arm entry of the EPM (median [IQR]; Veh: 95.2 [68.6–114.3], BHB: 60.0 [25.7–102.9], Mann–Whitney’s U-test; W = 54.5, p = 0.23). (d) Time spent in closed arms of the EPM (median [IQR]; Veh: 103.2 [101.4–103.2], BHB: 103.2 [101.0–103.2], Mann–Whitney’s U-test; W = 40.5, p > 0.99). (e) Time staying the center area of the EPM (median [IQR]; Veh: 0.0 [0.0–0.0], BHB: 0.0 [0.0–0.0], Mann–Whitney’s U-test; W = 40.5, p > 0.99). (f) The number of protected head dipping of the EPM (median [IQR]; Veh: 100.0 [100.0–126.3], BHB: 126.3 [0.0–189.5], Mann–Whitney’s U-test; W = 39.5, p = 0.96). (g) The number of non-protected head dipping of the EPM (median [IQR]; Veh: 0.0 [0.0–66.7], BHB: 66.7 [0.0–171.4], Mann–Whitney’s U-test; W = 33.5, p = 0.53). (h) The number of stretched-attend posture of the EPM (median [IQR]; Veh: 0.0 [0.0–171.4], BHB: 85.7 [0.0–150.0], Mann–Whitney’s U-test; W = 37.5, p = 0.82). (i) The number of grooming of the EPM (median [IQR]; Veh: 80.0 [40.0–160.0], BHB: 80.0 [54.5–160.0], Mann–Whitney’s U-test; W = 39.5, p = 0.97). (j) The number of rearing of the EPM (median [IQR]; Veh: 105.0 [82.5–127.5], BHB: 97.5 [94.3–120.0], Mann–Whitney’s U-test; W = 40.0, p > 0.99). (k) Time in the periphery area of the OFT (median [IQR]; Veh: 100.1 [98.0–102.8], BHB: 102.8 [100.8–102.8], Mann–Whitney’s U-test; W = 21.0, p = 0.073). (l) The number of center area of the OFT (median [IQR]; Veh: 112.5 [0.0–182.6], BHB: 0.0 [0.0–0.0], Mann–Whitney’s U-test; W = 59.0, p = 0.070). (m) Time spent in the center area of the OFT (median [IQR]; Veh: 85.7 [0.0–200.0], BHB: 0.0 [0.0–0.0], Mann–Whitney’s U-test; W = 58.5, p = 0.079). (n) Latency to the center area of the OFT (median [IQR]; Veh: 72.0 [39.5–156.5], BHB: 156.5 [156.5–156.5], Mann–Whitney’s U-test; W = 23.5, p = 0.12). (o) The number of rearing of the OFT (median [IQR]; Veh: 100.0 [29.0–173.8], BHB: 24.8 [20.0–78.6], Mann–Whitney’s U-test; W = 57.0, p = 0.16). (p) The number of grooming of the OFT (median [IQR]; Veh: 105.9 [69.2–115.4], BHB: 70.6 [35.3–115.4], Mann–Whitney’s U-test; W = 45.0, p = 0.72). Data are shown using % of controls. Reference control is the Veh group. The data are presented as scatter plots including median and interquartile range. Mann–Whitney’s U-test was performed to compare two groups. n = 9/group. EPM: elevated plus maze test, OFT: open field test, Veh: Vehicle (PBS), BHB: beta-hydroxybutyrate, n = 9. **Supplemental Figure 4**: The numbers of animal used in this study. Totally, 125 rats were used in this study. Rats were randomly divided into each experimental group randomly without any consideration of baseline information.

Supplementary Information 2.

Supplementary Information 3.

## Data Availability

Raw data are shown in Supplemental Tables.
