# Supplementary material for: Beta-hydroxybutyrate, an endogenous NLRP3 inflammasome inhibitor, attenuates anxiety-related behavior in a rodent post-traumatic stress disorder model
Source: Sci Rep. 2020 Dec 10;10:21629. doi: 10.1038/s41598-020-78410-2 (PMC7728809; doi:10.1038/s41598-020-78410-2)
Supplement: Supplementary file 1 — Supplementary Information 1. Supplemental Figure 1: Other behavior comparisons between non-SPS rats and SPS rats. (a) The numbers of closed arms entry of the EPM (median [IQR]; non SPS: 104.1 [87.3–131.7], SPS: 140.5 [110.1–164.7], Mann–Whitney’s U-test; W = 42.5, p = 0.094). (b) The numbers of staying center area of the EPM (median [IQR]; non SPS: 97.5 [45.0–127.5], SPS: 0.0 [0.0–86.3], Mann–Whitney’s U-test; W = 101, p = 0.082). (c) The numbers of open and closed arm entry of the EPM (median [IQR]; non SPS: 109.7 [76.7–130.6], SPS: 105.0 [74.8–132.4], Mann–Whitney’s U-test; W = 70, p = 0.93). (d) Time spent in closed arms of the EPM (median [IQR]; non SPS: 98.5 [82.7–117.9], SPS: 125.6 [118.4–128.9], Mann–Whitney’s U-test; W = 30.5, p = 0.018). (e) Time staying the center area of the EPM (median [IQR]; non SPS: 98.5 [18.75–118.0], SPS: 0.0 [0.0–59.4], Mann–Whitney’s U-test; W = 95.5, p = 0.16). (f) The number of protected head dipping of the EPM (median [IQR]; non SPS: 92.1 [47.7–150.0], SPS: 129.6 [102.3–262.5], Mann–Whitney’s U-test; W = 48.5, p = 0.18). (g) The number of non-protected head dipping of the EPM (median [IQR]; non SPS: 109.6 [17.3–141.4], SPS: 40.4 [0.0–69.2], Mann–Whitney’s U-test; W = 96.5, p = 0.16). (h) The number of stretched-attend posture of the EPM (median [IQR]; non SPS: 50.0 [0.0–200.0], SPS: 175.0 [137.5–525.0], Mann–Whitney’s U-test; W = 35, p = 0.033). (i) The number of grooming of the EPM (median [IQR]; non SPS: 120.0 [0.0–171.4], SPS: 120.0 [64.3–244.3], Mann–Whitney’s U-test; W = 57, p = 0.39). (j) The number of rearing of the EPM (median [IQR]; non SPS: 104.1 [65.5–116.9], SPS: 128.0 [122.2–156.4], Mann–Whitney’s U-test; W = 32, p = 0.022). (k) Time in the periphery area of the OFT (median [IQR]; non SPS: 101.0 [98.8–101.2], SPS: 101.0 [97.6–101.7], Mann–Whitney’s U-test; W = 72, p > 0.99). (l) The number of center area of the OFT (median [IQR]; non SPS: 58.4 [0.0–158.3], SPS: 79.6 [12.5–255.7], Mann–Whitney’s U-test; W = 66, p = 0 [file 41598_2020_78410_MOESM1_ESM.pptx]

## Slide 1
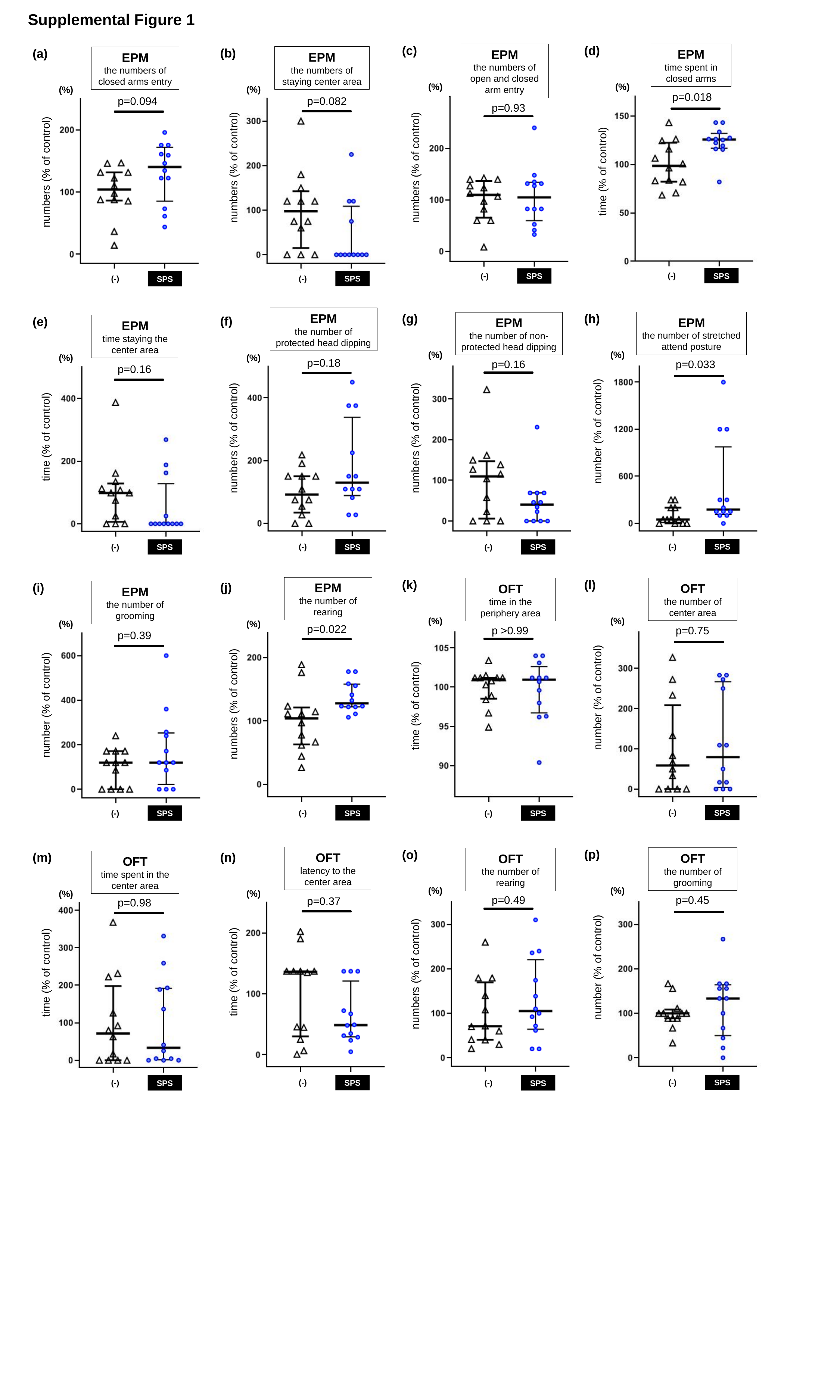

Supplemental Figure 1
(d)
(c)
(b)
(a)
EPM
time spent in closed arms
EPM
the numbers of open and closed arm entry
EPM
the numbers of staying center area
EPM
the numbers of closed arms entry
(%)
(%)
(%)
(%)
p=0.018
p=0.094
p=0.082
p=0.93
time (% of control)
numbers (% of control)
numbers (% of control)
numbers (% of control)
(-)
(-)
SPS
SPS
(-)
(-)
SPS
SPS
(h)
EPM
the number of protected head dipping
(g)
(f)
(e)
EPM
the number of stretched attend posture
EPM
the number of non-protected head dipping
EPM
time staying the center area
(%)
(%)
(%)
(%)
p=0.18
p=0.16
p=0.033
p=0.16
time (% of control)
number (% of control)
numbers (% of control)
numbers (% of control)
(-)
(-)
SPS
(-)
SPS
(-)
SPS
SPS
(l)
(k)
(j)
(i)
EPM
the number of rearing
OFT
the number of center area
OFT
time in the periphery area
EPM
the number of grooming
(%)
(%)
(%)
(%)
p=0.022
p >0.99
p=0.75
p=0.39
number (% of control)
time (% of control)
number (% of control)
numbers (% of control)
(-)
(-)
SPS
(-)
SPS
(-)
SPS
SPS
(p)
(o)
(n)
(m)
OFT
latency to the center area
OFT
the number of grooming
OFT
the number of rearing
OFT
time spent in the center area
(%)
(%)
(%)
(%)
p=0.49
p=0.45
p=0.37
p=0.98
time (% of control)
time (% of control)
number (% of control)
numbers (% of control)
(-)
(-)
SPS
(-)
SPS
(-)
SPS
SPS

## Slide 2
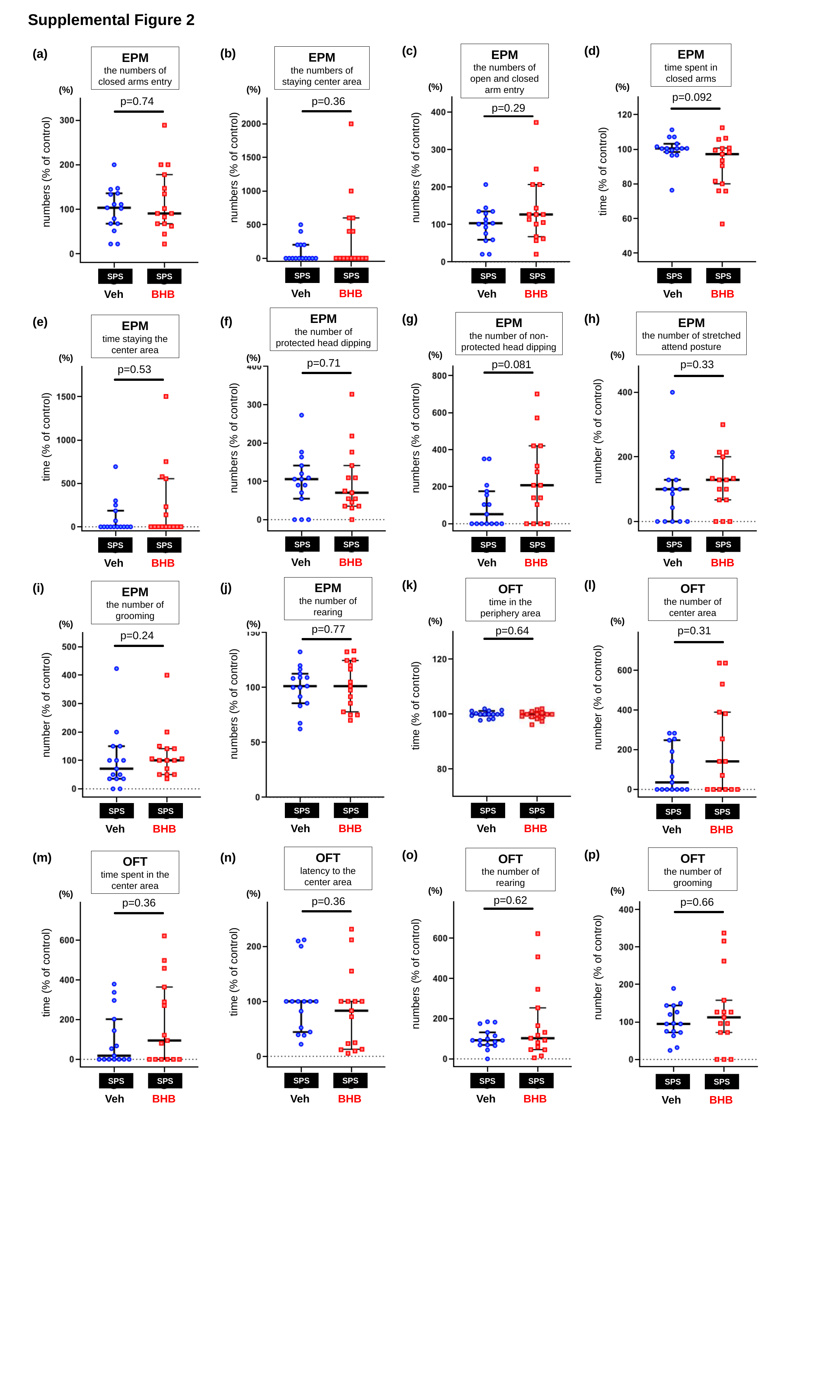

Supplemental Figure 2
(d)
(c)
(b)
(a)
EPM
time spent in closed arms
EPM
the numbers of open and closed arm entry
EPM
the numbers of staying center area
EPM
the numbers of closed arms entry
(%)
(%)
(%)
(%)
p=0.092
p=0.74
p=0.36
p=0.29
time (% of control)
numbers (% of control)
numbers (% of control)
numbers (% of control)
SPS
SPS
SPS
SPS
SPS
SPS
SPS
SPS
Veh
BHB
Veh
BHB
BHB
Veh
BHB
Veh
(h)
EPM
the number of protected head dipping
(g)
(f)
(e)
EPM
the number of stretched attend posture
EPM
the number of non-protected head dipping
EPM
time staying the center area
(%)
(%)
(%)
(%)
p=0.71
p=0.081
p=0.33
p=0.53
time (% of control)
number (% of control)
numbers (% of control)
numbers (% of control)
SPS
SPS
SPS
SPS
SPS
SPS
SPS
SPS
Veh
BHB
Veh
BHB
BHB
Veh
BHB
Veh
(l)
(k)
(j)
(i)
EPM
the number of rearing
OFT
the number of center area
OFT
time in the periphery area
EPM
the number of grooming
(%)
(%)
(%)
(%)
p=0.77
p=0.64
p=0.31
p=0.24
number (% of control)
time (% of control)
number (% of control)
numbers (% of control)
SPS
SPS
SPS
SPS
SPS
SPS
SPS
SPS
Veh
BHB
BHB
Veh
BHB
Veh
Veh
BHB
(p)
(o)
(n)
(m)
OFT
latency to the center area
OFT
the number of grooming
OFT
the number of rearing
OFT
time spent in the center area
(%)
(%)
(%)
(%)
p=0.62
p=0.36
p=0.66
p=0.36
time (% of control)
time (% of control)
number (% of control)
numbers (% of control)
SPS
SPS
SPS
SPS
SPS
SPS
SPS
SPS
Veh
BHB
BHB
Veh
BHB
Veh
Veh
BHB

## Slide 3
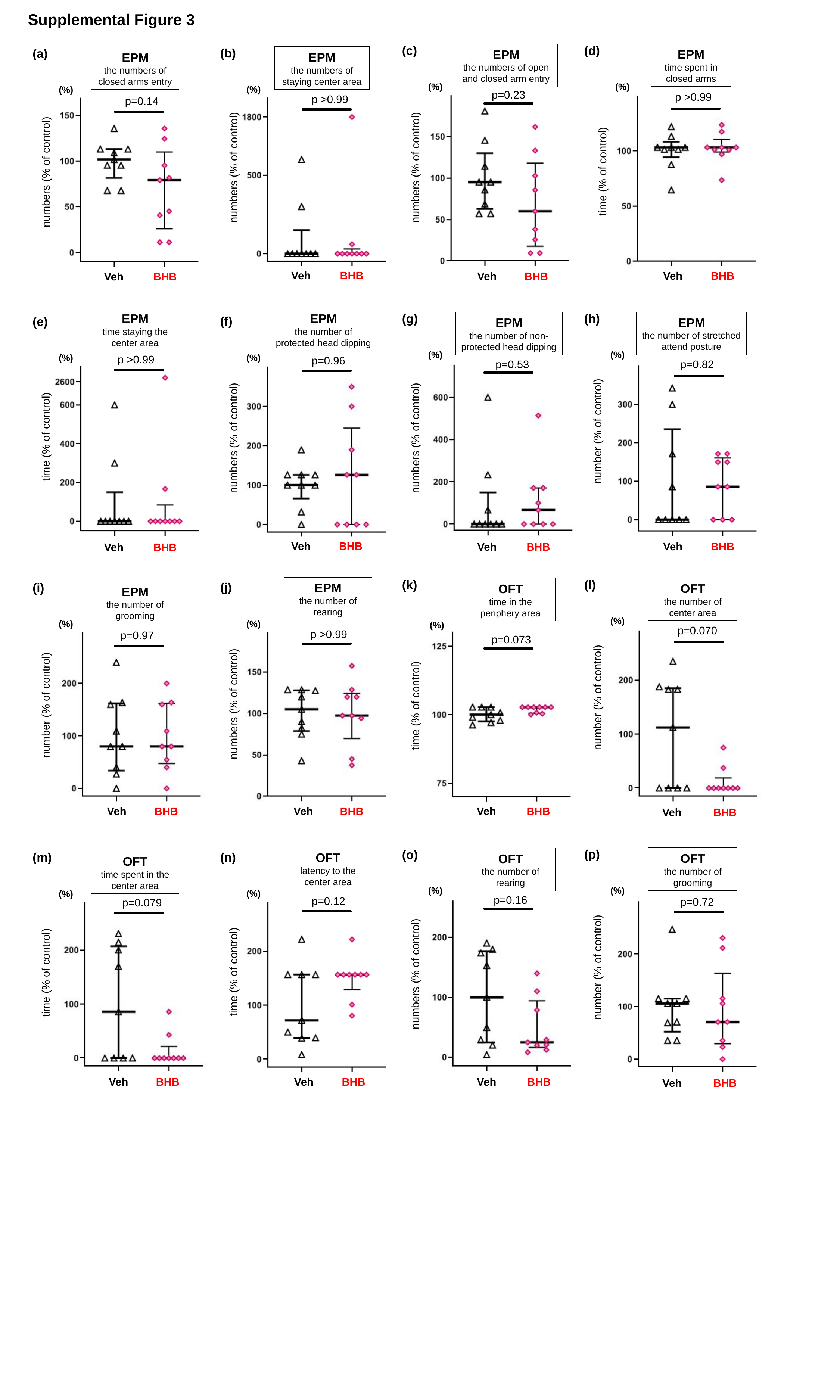

Supplemental Figure 3
(d)
(c)
(b)
(a)
EPM
time spent in closed arms
EPM
the numbers of open and closed arm entry
EPM
the numbers of staying center area
EPM
the numbers of closed arms entry
(%)
(%)
(%)
(%)
p=0.23
p >0.99
p >0.99
p=0.14
time (% of control)
numbers (% of control)
numbers (% of control)
numbers (% of control)
Veh
BHB
Veh
BHB
BHB
Veh
BHB
Veh
(h)
EPM
time staying the center area
EPM
the number of protected head dipping
(g)
(f)
(e)
EPM
the number of stretched attend posture
EPM
the number of non-protected head dipping
(%)
(%)
(%)
(%)
p >0.99
p=0.96
p=0.53
p=0.82
time (% of control)
number (% of control)
numbers (% of control)
numbers (% of control)
Veh
BHB
Veh
BHB
BHB
Veh
BHB
Veh
(l)
(k)
(j)
(i)
EPM
the number of rearing
OFT
the number of center area
OFT
time in the periphery area
EPM
the number of grooming
(%)
(%)
(%)
(%)
p=0.070
p >0.99
p=0.97
p=0.073
number (% of control)
time (% of control)
number (% of control)
numbers (% of control)
Veh
BHB
BHB
Veh
BHB
Veh
Veh
BHB
(p)
(o)
(n)
(m)
OFT
latency to the center area
OFT
the number of grooming
OFT
the number of rearing
OFT
time spent in the center area
(%)
(%)
(%)
(%)
p=0.16
p=0.12
p=0.72
p=0.079
time (% of control)
time (% of control)
number (% of control)
numbers (% of control)
Veh
BHB
BHB
Veh
BHB
Veh
Veh
BHB

## Slide 4
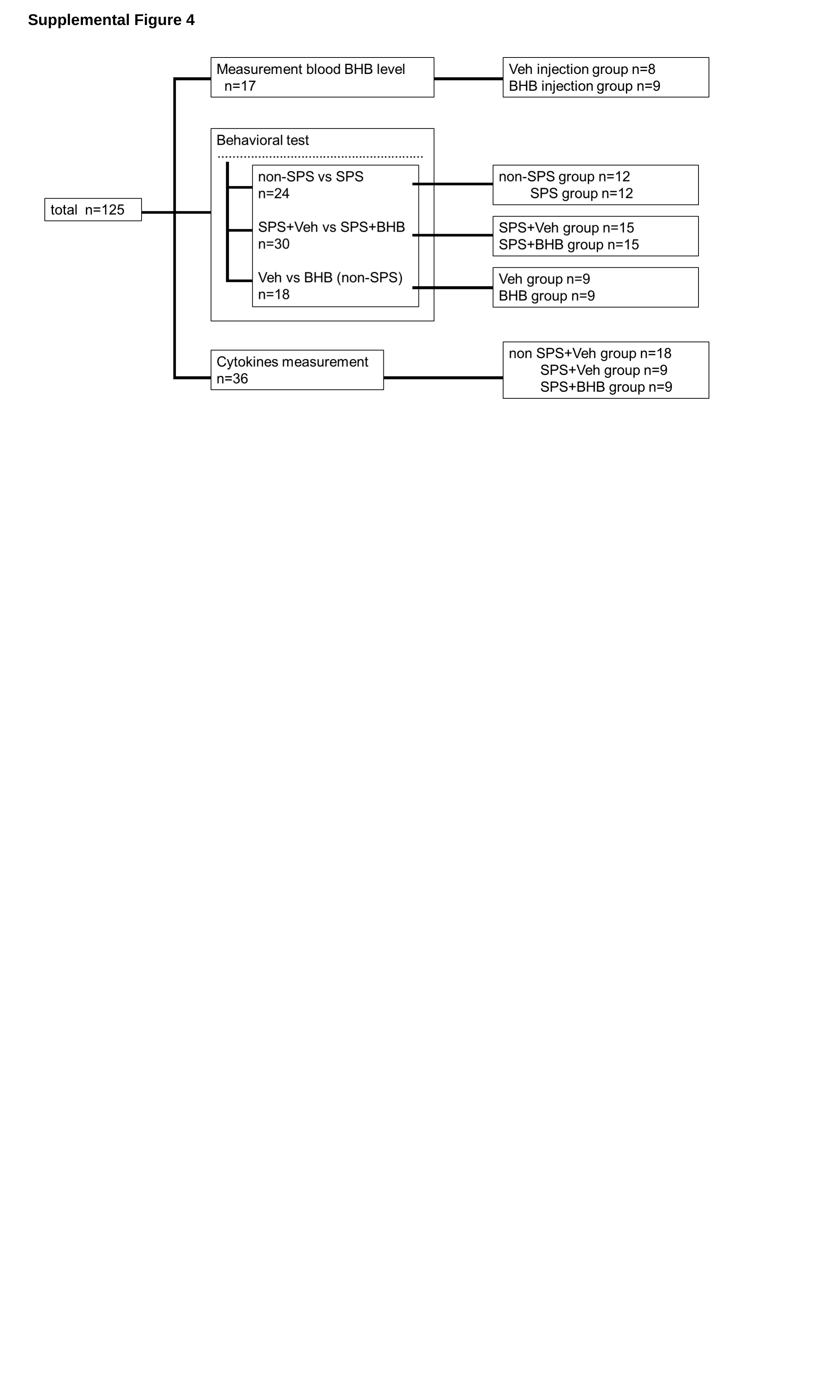

Supplemental Figure 4
